# Mechanisms of hepatic and renal injury in lipid metabolism disorders in metabolic syndrome

**DOI:** 10.7150/ijbs.100394

**Published:** 2024-09-09

**Authors:** Jin Rong, Zixuan Zhang, Xiaoyu Peng, Ping Li, Tingting Zhao, Yifei Zhong

**Affiliations:** 1 Institute of Clinical Medical Sciences, State Key Laboratory of Respiratory Health and Multimorbidity, China-Japan Friendship Hospital, Beijing, PR China.; 2 Department of Nephrology A, Longhua Hospital Shanghai University of Traditional Chinese Medicine, Shanghai, PR China.; 3 College of Life Science and Technology, Shandong Second Medical University, Weifang, Shandong, PR China.; 4 College of Life Science and Technology, Beijing University of Chemical Technology, Beijing, PR China.

**Keywords:** Metabolic Syndrome, Diabetic Kidney Disease, Non-alcoholic Fatty Liver Disease, Lipid Metabolism, Treatment

## Abstract

Metabolic syndrome (MetS) is a group of metabolic abnormalities that identifies people at risk for diabetes and cardiovascular disease. MetS is characterized by lipid disorders, and non-alcoholic fatty liver disease (NAFLD) and diabetic kidney disease (DKD) are thought to be the common hepatic and renal manifestations of MetS following abnormal lipid metabolism. This paper reviews the molecular mechanisms of lipid deposition in NAFLD and DKD, highlighting the commonalities and differences in lipid metabolic pathways in NAFLD and DKD. Hepatic and renal steatosis is the result of lipid acquisition exceeding lipid processing, i.e., fatty acid uptake and lipid regeneration exceed fatty acid oxidation and export. This process is directly regulated by the interactions of nuclear receptors, transporter proteins and transcription factors, whereas pathways such as oxidative stress, autophagy, cellular pyroptosis and gut flora are also key regulatory hubs for lipid metabolic homeostasis but act slightly differently in the liver and kidney. Such insights based on liver-kidney similarities and differences offer potential options for improved treatment.

## Introduction

Metabolic syndrome (MetS) is a group of abnormal metabolic syndromes characterised by the aggregated onset of obesity, dyslipidaemia, hyperglycaemia and hypertension. Insulin resistance (IR) is considered to be the core of MetS, and visceral or intra-abdominal adipose tissue levels are the main component and initialising factor of the metabolic syndrome 1, which induces the development of IR, followed by a series of metabolic abnormalities and clinical symptoms in the body. MetS is a global epidemic, posing a serious risk to human health and public health. The prevalence of metabolic syndrome varies according to criteria defined by different organizations, and it is estimated that metabolic syndrome occurs in approximately one quarter of the world's population 2. According to estimates from the Centers for Disease Control and Prevention, the prevalence of MetS stands at 34.7% in the United States 3 and 33.9% in China 4. Over recent decades, there has been a marked increase in severity, particularly among individuals over 60 years old, driven by rising rates of diabetes and obesity. Moreover, there is an annual growth rate exceeding 8% in this age group 3, 5. The liver and kidney are the two major organs that maintain metabolic homeostasis in the body, and both respond synergistically to changes in nutrient availability and energy requirements. In recent years, some studies have found MetS-related liver and kidney damage, indicating that MetS is closely related to the decline in renal function and the progression of diabetic kidney disease (DKD), and the typical pathological change of MetS involving the liver is non-alcoholic fatty liver disease (NAFLD), whose prevalence increasing trend is in line with that of the prevalence of obesity and metabolic disorders 1, 6.

**Figure 1:**
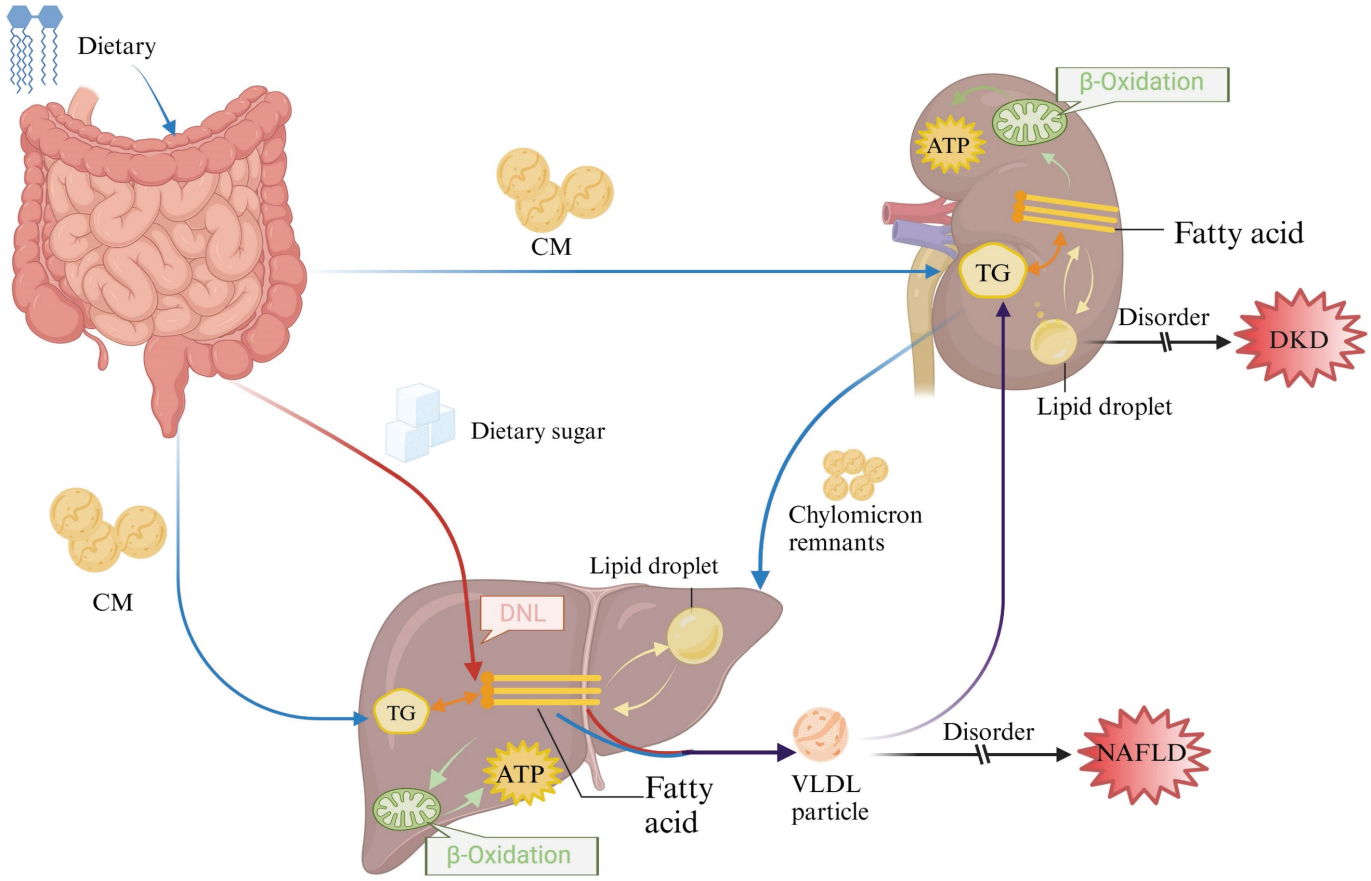
Overview of lipid metabolism in the liver and kidney. After dietary digestion in the gut, part of the lipids are utilized within the intestine, while the remaining lipids are packaged into chylomicrons and transported to peripheral tissues and other organs (depicted by the blue arrow). Simultaneously, sugars absorbed in the gut undergo *de novo* synthesis in the liver, leading to the synthesis of endogenous lipids (depicted by the red arrow). These endogenous lipids, mixed with exogenous lipids (illustrated by the purple arrow), are transported to the kidneys and other tissues in the form of VLDL for absorption and utilization**.**CM: chylomicrons; DNL: *De novo* lipogenesis; TG: Triglyceride.

Lipid disorders are one of the major risk factors for MetS. Studies have shown that obesity and lipid-related parameters are highly correlated with metabolic syndrome and can be used to predict MetS, with lipid accumulation products showing the best predictive results 7. Dietary triglycerides and cholesterol esters are released in the form of chylomicrons (CM), whereas internally synthesized fatty acids from the liver are either released or stored in lipid droplets within very low-density lipoproteins (VLDL) 8, 9. The circulating CM and VLDL particles are absorbed by organs such as the kidneys. As a high energy consumption organ, kidney tubular cells derive 90% of their energy from fatty acids 10 and then the remaining chylomicron residue is delivered to the liver (Fig 1). Lipid metabolism disorders can trigger the development of atherosclerosis through the activation of oxidative stress and inflammatory responses, thus promoting the development of cardiovascular disease 11. In recent years, disorders of lipid metabolism have been found to be involved in the development of DKD, as well as a major factor linking DKD to other organs or diseases. Clinical evidence suggests that non-alcoholic steatohepatitis (NASH) is an independent risk factor for chronic kidney disease (CKD), which has been phenotypically validated for CKD in a mouse model of MASH that mimics human NASH, and alterations in the lipid profile of the renal cortex mediated by NASH have been found 12, which suggests that the critical influence of abnormal lipid metabolism in the disease process of NAFLD and DKD as well as in liver-kidney interactions. Given the widespread global prevalence of metabolic syndrome, the current consensus on the key pathogenic mechanisms of NAFLD and DKD and their progression is evolving. This article provides an overview of the potential mechanisms, regulatory pathways, and treatments related to how lipid metabolism disorders can contribute to or worsen the progression of NAFLD and DKD.

## Lipid metabolism in the liver and kidney

Lipid metabolism in the liver and kidney comprises four primary processes: uptake of circulating fats, lipid synthesis, fatty acid oxidation, and cholesterol efflux. In pathological states, dysregulation of lipid metabolism leads to increased production of circulating free fatty acids and their deposition in non-adipose tissues such as the liver and kidneys. This ectopic deposition eventually becomes the causative factor in the development of NAFLD and DKD. However, the liver is the central mediator of lipid metabolism, and the kidney is an important organ for lipid filtration. NAFLD and DKD that begin with lipid metabolism disorders will aggravate liver and kidney injuries with the course of the disease, thus affecting the synthesis, transport, and catabolism of lipids, and at the same time interfering with the filtration and metabolism of lipids in the kidney, disrupting the balance of hormones and enzymes related to lipid metabolism, and exacerbating the disorders of lipid metabolism. The relationship between disorders of lipid metabolism and hepatic and renal injury is therefore bidirectional, and the interplay between the two often needs to be considered together in the treatment and management of these diseases.

### Increased uptake of circulating lipids

Fatty acids serve as energy reserves in adipocytes and are transported to energy-demanding tissues by a group of proteins proposed to facilitate the translocation of fatty acids includes the six-member fatty acid transport protein (FATP), cluster of differentiation 36 (CD36), and fatty acid-binding proteins (FABP), which regulate lipid flux, transport, signal transduction, and metabolism. These proteins play essential roles in lipid metabolism in the liver and kidneys at different stages of fatty acid uptake 13.

FATP was first identified by Schaffer *et al.* in 3T3-L1 adipocytes from mice. It plays a crucial role in fatty acid uptake and primarily functions by esterifying coenzyme A during activation 13. There are six isoforms of FATP in mammals, of which FATP2, FATP4 and FATP5 play major regulatory roles in hepatic and renal lipid metabolism. In the liver and kidney, FATP2 and FATP4 have been shown to be the main cause of abnormal lipid uptake in patients with metabolic liver and kidney disease 14. Knockdown of FATP2 and/or FATP4 in mice reduces fatty acid uptake in the liver while ameliorating steatosis. FATP2 deletion reduces proteinuria and restores glomerular filtration rate to normal in obese mice. FATP4 expression can be increased in renal tubules of mice fed a high-fat diet 15. Although FATP2 and FATP4 are both highly expressed in the liver and kidney, they differ in that FATP2 and FATP4 are mainly responsible for the transport of free fatty acids in the tubules in the kidney, and mainly regulate the lipid uptake of proximal renal tubular cells in the high-glucose environment and the activity of fatty acid oxidases to mediate the onset and progression of DKD 16. In the liver, FATP2 and FATP4 are highly expressed in hepatocytes and actively carry out extracellular free fatty acid uptake, and after exerting very long-chain acyl-coenzyme A synthetase activity, a gradient difference in concentration of free fatty acids is formed between the inside and outside of the cell, thus enabling transmembrane transport of fatty acid 17. Similar to FATP2 and FATP4, FATP5, which also increases cellular uptake of fatty acids, is also abundantly expressed in the liver. Knockdown of FATP5 in the liver decreased cellular uptake of long-chain fatty acids and also reversed diet-induced hepatic steatosis in mice 18. There is a paucity of studies in which FATP5 is associated with metabolic nephropathy, and more studies are needed to expand our current understanding of the role of FATP5 in clinical DKD.

CD36 acts as a high-affinity scavenger receptor on the cell surface, and its membrane suggests an open-ended α-helical structure that attracts long-chain fatty acids and oxidised low-density lipoproteins (Ox-LDL) to attach to the CD36 membrane surface. Subsequently, CD36 is palmitoylated and modified to localise to the cell membrane to bind fatty acids 19. CD36 plays a key role in the uptake of free fatty acids by the liver and kidney. For the liver and kidney, CD36 is responsible for the uptake of free fatty acids and the transport of the resulting free fatty acids into hepatocytes and podocytes, respectively. Previous research has shown that abnormal CD36 levels play a causal role in the development of steatosis in patients with DKD and NAFLD, with CD36-deficient mice exhibiting lower hepatic and renal lipid accumulation and hepatic and renal injury compared to controls 20.

FABP functions as an intracellular carrier for fatty acids, crucially regulating their uptake, transport, and metabolism through reversible binding within cells. This process contributes to abnormal lipid uptake observed in the liver and kidney. FABP is classified into nine subtypes according to tissue specificity. Liver-type FABP (L-FABP; also known as FABP1), adipocyte FABP (A-FABP; also known as FABP4), and epidermal-type FABP (E-FABP; also known as FABP5) are all expressed in liver and kidney 21. L-FABP facilitates fatty acid and acyl CoA transport, storage, and utilization. It may protect against lipotoxicity by binding cytotoxic free fatty acids, promoting oxidation, or incorporating them into triglycerides. Deletion of L-FABP in animals increases E-FABP expression and cholesterol accumulation 22. In addition, FABPs also promote polyunsaturated fatty acid-induced transcriptional activation of PPARα and PPARγ through direct interactions with the ligand-binding domains of nuclear receptors 23. Among them, A-FABP levels were found to be significantly higher in early stage of DKD patients and in NAFLD patients and were negatively correlated with glomerular filtration function and positively correlated with percentage of liver fat 24. The expression levels of L-FABP and E-FABP in NAFLD patients and their effects on liver lipids have been shown to be similar to those of A-FABP 25. L-FABP is mainly expressed in the liver and only a small amount is expressed in the kidney. Under physiological conditions, liver-derived L-FABP is released into the blood circulation, filtered through the glomerulus and reabsorbed into the renal tubules. In a rat model of nephropathy, high levels of L-FABP in the urine promote the proliferation of tubular epithelial cells in the rat, which can exacerbate interstitial inflammation 26. Based on its important role in fatty acid uptake and cell signalling, urinary L-FABP is considered to be one of the potential markers of disease progression in DKD 27. Although E-FABP has also been suggested to have the ability to promote fatty acid uptake in podocytes, its mechanism of action in renal disease is unclear and requires further study.

### Increased lipid synthesis and deposition

Lipid synthesis in hepatocytes and podocytes is closely linked to glucose levels. With elevated glucose in the circulatory system, glycolytic reactions responsible for glucose metabolism are initiated, leading to the production of pyruvate, which is subsequently translocated into the mitochondria to be converted to citric acid. Citric acid can be used directly as a carbon source for fatty acids through ATP citrate lyase (ACLY) and denaturation regulation, controlling fatty acid anabolic reactions 28. As a result, free fatty acids are not degraded but are shuttled to form triglycerides secreted as very low-density lipoproteins. Insulin and glucose regulation involves transcription factors sterol regulatory element-binding protein 1c (SREBP1c) and carbohydrate response element binding protein (ChREBP), which play pivotal roles in controlling glycolysis and lipid synthesis in the hepatocytes and podocytes 29, 30. Both SREBP-1c and ChREBP enhance the synthesis of crucial enzymes involved in glycolysis and lipogenesis, such as acetyl Coenzyme A carboxylase (ACC) and fatty acid synthase (FASN) 31. This leads to increased triglyceride levels, thereby playing a significant role in the onset of metabolic disorders like DKD and NAFLD.

In glycolipid metabolism, ChREBP plays a significant role in DKD by primarily regulating inflammation and renal fibrosis. Elevated ChREBP levels have been associated with increased serum levels of various inflammatory cytokines 32. It was found that in the kidney, ChREBP was highly expressed in renal proximal tubular cells, and activation of ChREBP in renal proximal tubular cells mediated a significant elevation of hypoxia-inducible factor-1α (HIF-1α), inducing glomerulosclerosis and tubulointerstitial injury 30, 33, 34. The above suggests that ChREBP-mediated lipid accumulation is involved in the development of renal injury in DKD, and the mechanism may be related to the activation of NLRP3 inflammatory vesicles 35. In addition to renal proximal tubule cells, ChREBP is also highly expressed in hepatocytes, with the exception of the regulation of glycogen homeostasis and hepatic steatosis, may also have an important role in counteracting the hepatotoxicity induced by the high fructose diet (HFrD). Knockdown of ChREBP prevents fructose-induced steatosis in mice, but ChREBP systemic knockout mice are intolerant to HFrD, have reduced expression of fat synthases such as fatty acid synthase, increased glycogen content, and develop features of liver injury 36. Therefore, there may be a protective mechanism of ChREBP on the liver: HFrD induces ChREBP expression and promotes the elevation of fibroblast growth factor in intrahepatocytes and plasma, which reduces hepatic inflammation 37; ChREBP can also inhibit cholesterol over-synthesis by reducing SREBP2 expression 38. In addition, excessive fructose intake leads to uncontrolled ATP depletion and accumulation of intermediary metabolites, and ChREBP may maintain ATP homeostasis by activating the target gene LPK at high glucose to attenuate ATP imbalance-induced liver injury 39.

### Decreased fatty acid oxidation

Fatty acid oxidation mainly occurs in mitochondria, particularly when glucose levels are low, providing crucial ATP for hepatic and renal cortical tissues. Peroxisome proliferator-activated receptor α (PPARα) activation upregulates genes like acyl-CoA oxidase, peroxisome proliferator-activated receptor-gamma coactivator-1alpha (PGC-1α), and carnitine palmitoyltransferase 1 (CPT1), supporting lipid homeostasis in the liver and kidney 40. Among them, PGC-1α promotes mitochondrial energy homeostasis and oxidative metabolism, while up-regulating the expression of CPT1, which acts as a rate-limiting enzyme for fatty acid β-oxidation and is located in the outer membrane of mitochondria, and transfers long-chain fatty acyl-CoA to carnitine for translocation into mitochondria for further oxidation, and is a key regulator enzyme in maintaining the homeostasis of fatty acid metabolism 41. It has been demonstrated that dysregulation of PPARα expression levels and its associated signalling pathways can lead to impaired fatty acid oxidation in the liver and kidney, inducing NAFLD with DKD 42. PGC-1α, as a co-stimulator of PPARα, can stimulate the synthesis of mitochondrial enzymes, improve mitochondrial function, promote the decomposition of fatty acids in the podocytes of DKD patients, and at the same time reduce the accumulation of triglycerides, alleviating hepatic steatosis in NAFLD patients 43. The high expression of CPT1 in proximal tubular cells of liver and renal tubules also has a profound effect on the control of fatty acid oxidation and cellular energy homeostasis, accelerating fatty acid β-oxidation and promoting lipid metabolism 44.

The peroxisome is vital for lipid balance in the liver and kidney, with distinct regulatory mechanisms. Fatty acids can be oxidised in the peroxisome. The first and rate-limiting step of peroxisomal β-oxidation is carried out by the family of acyl coenzyme A oxidase proteins (ACOX1, ACOX2, and ACOX3), of which ACOX1 is enriched in the liver. During fatty acid β-oxidation in peroxisomes, ACOX1 catalyses the desaturation of acyl coenzyme a to produce 2-trans-enyl coenzyme a generating hydrogen peroxide (H_2_O_2_) as a by-product 45. The peroxidase body contains catalase, which rapidly breaks down H_2_O_2_ into water and oxygen in response to changes in cellular energy and metabolic demands, thereby reducing unnecessary fatty acid oxidation and potential damage to hepatocytes. Succinate, the central pathogenic molecule in DKD, is produced via peroxisomal dicarboxylic acid metabolism. It inhibits renal mitochondrial lipid metabolism in DKD mice, leading to overproduction of reactive oxygen species 46. This in turn causes significant accumulation of renal lipids and oxidative stress, ultimately leading to glomerulopathy. Excess succinate can also lead to abnormally high blood pressure by activating the renin-angiotensin-aldosterone system 47. Reducing peroxisomal succinate production may protect proximal renal tubular cells and minimise kidney damage in patients with DKD.

### Cholesterol efflux dysregulation

Cholesterol efflux is vital for reverse cholesterol transport, moving cholesterol from peripheral tissues to the liver and kidneys. Dysfunctions lead to free cholesterol accumulation, driving lipotoxicity, necroinflammation, and fibrosis. These contribute to NAFLD and DKD progression 48-50. This process is mainly mediated by ATP-binding cassette transporter protein A1 (ABCA1) and liver X receptor (LXR).

ABCA1 facilitates cholesterol and phospholipid transport to apoA-I, forming new HDL particles that enter circulation. LXRα, a key regulator of ABCA1, activates its expression, promoting cholesterol efflux, reducing foam cell formation and lipid aggregation. This inhibits NAFLD and DKD development 51, 52. LXR belongs to the nuclear receptor superfamily and exists in two isoforms: LXRα (NR1H3) and LXRβ (NR1H2). LXRβ is widely expressed in a variety of tissues, whereas LXRα is specifically expressed in organs such as the liver, kidney and intestine. LXRα acts as an important transcription factor forming a heterodimer with the retinoid X-like receptor, which binds to the DR4 element in the promoter region of the ABCA1 gene 53. Increased levels of oxysterols derived from intracellular cholesterol promote the transcriptional function of LXR, resulting in heightened ABCA1 expression. This process facilitates reverse cholesterol transport, playing a vital role in regulating glycolipid metabolism and maintaining cholesterol homeostasis 54. In addition, LXR was coupled with the PPARγ pathway to regulate lipid metabolism, thereby exerting anti-NAFLD and DKD effects 55.

Clinical studies have found that ABCA1 levels in both NAFLD and DKD patients are significantly lower than in healthy patients, and that reduced ABCA1 expression is positively correlated with markers of progression in NAFLD and DKD 56-58. When ABCA1 function is lost, cholesterol efflux is disrupted, resulting in the accumulation of cholesterol in the liver and kidneys 59. Overexpression of ABCA1 promotes increased cholesterol efflux in both hepatocytes and podocytes and decreased lipid accumulation is reduced. In addition, abnormally high levels of LXRα expression were observed in NAFLD hepatocytes and DKD renal tubular cells, while LXRβ expression was unchanged 60.

## Pathway of lipid metabolism in liver and kidney

**Figure 2:**
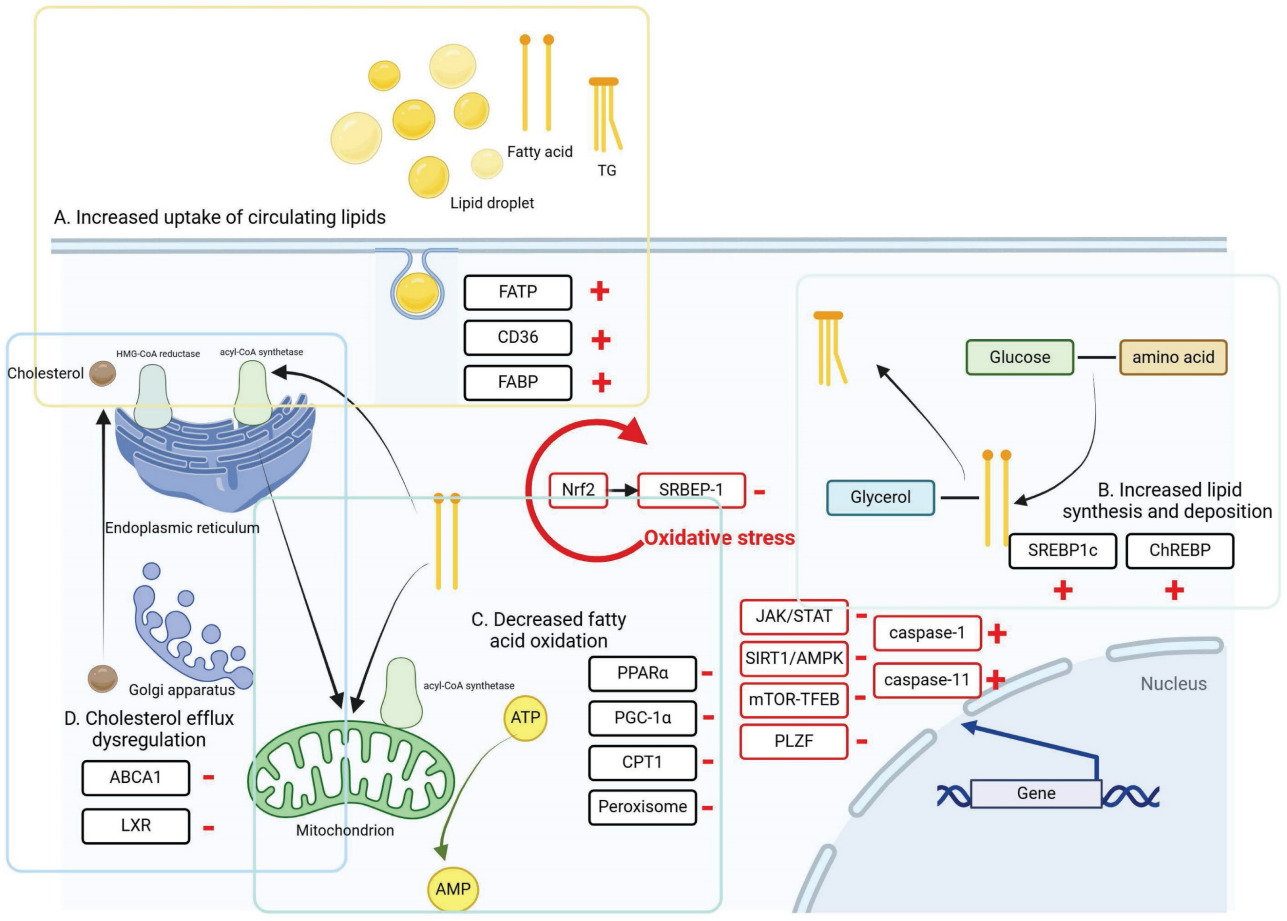
The intracellular mechanisms and regulatory pathways of liver and kidney injury caused by lipid metabolism disorders. During lipid metabolism disorders, ccells regulate intracellular lipid accumulation and lipid peroxidation through oxidative stress, autophagy, and cellular pyroptosis, thereby alleviating lipid deposition in the liver and kidneys.

There are several signaling pathways involved in hepatic and renal lipid metabolism, which can be broadly categorized into oxidative stress, autophagy and cellular pyroptosis. These processes are directly regulated by interactions among nuclear receptors, transporter proteins, and transcription factors, illustrated in Figure 2. In recent years, growing evidence has emphasized the significant impact of gut microbiota on regulating host lipid metabolism. These mechanisms are discussed in this section.

### Regulation of oxidative stress

Under normal physiological conditions, the body maintains a delicate balance between oxidative and antioxidant mechanisms. However, any disruption in this equilibrium can lead to oxidative stress, characterized by the overproduction of reactive oxygen species (ROS). This imbalance can subsequently induce various irregularities in lipid metabolism, such as lipid peroxidation 61, 62. Lipid peroxidation products are deposited in hepatocytes, glomerular basement membranes, and tubular interstitium, causing liver and kidney damage and leading to the development of NAFLD and DKD 63-66. Meanwhile, lipid peroxidation, as a highly reactive compound, can itself further generate ROS, resulting in a vicious cycle. Nuclear factor E2-related factor 2 (Nrf2) serves as a pivotal signaling hub connecting oxidative stress to the promotion of lipid buildup in adipose tissue. In adipocytes, Nrf2 modulates oxidative stress-triggered lipid accumulation by upregulating lipogenesis while downregulating lipolysis. Under oxidative stress conditions, Nrf2 recruitment to the SRBEP-1 promoter is enhanced, augmenting SREBP-1-mediated adipogenesis, and simultaneously inhibiting adipocyte lipolysis via the PKA pathway 67, 68*.*

### Regulation of autophagy

Autophagy-mediated lipid degradation is a crucial pathway for maintaining lipid homeostasis in the liver and kidney. When autophagy levels are insufficient to promptly clear damaged organelles like mitochondria, harmful substances can be released, disrupting cellular balance. This disruption can lead to oxidative stress, inflammation, and cytotoxicity, thereby worsening liver and kidney injuries 69.

The JAK/STAT signaling pathway has been demonstrated to attenuate lipid deposition and reverse palmitate-induced lipotoxicity in NAFLD and DKD by mediating hepatic and renal fat autophagy and reducing endoplasmic reticulum stress 70, 71. Lipid droplets store neutral lipids and release fatty acids for energy during nutrient scarcity, helping prevent lipotoxic cell damage in autophagy 72. This process, known as lipophagy, involving the selective lysosomal degradation of autophagophores within lipid droplets, is crucial for maintaining liver and kidney homeostasis by breaking down lipid droplets into free fatty acids 73. The study revealed that in liver samples diagnosed with NAFLD, there was suppression of the phagocytic function of fat, which closely correlated with disease progression in NAFLD patients 74, 75. Defects in autophagosome/lysosome fusion have been observed in the livers of mice fed a high-fat diet 76. In tubular cells of DKD patients and db/db mice, a deficiency in lipophagy has been observed, accompanied by significant ectopic lipid deposition 77. SIRT1, a member of the Sirtuins family of longevity proteins, is mainly found in the nucleus and is most closely related to autophagy regulation 78. For NAFLD and DKD, SIRT1 reduces fat accumulation in cells by regulating autophagy in hepatocytes and proximal renal tubular cells, respectively 79. Adenosine-phosphate-activated protein kinase (AMPK) plays a crucial role in regulating energy metabolism in the body. AMPK, which can be activated by various substances in the body, is a kinase that regulates autophagy through autophagy-related protein 1. Under adequate nutrition, the mTOR signaling pathway can inhibit lipid autophagy, mTOR can bind to other proteins to form mTOR complex 1 and mTOR complex 2, which play the role of serine and threonine protein kinase to promote autophagosome-lysosome fusion, regulate autophagy of fat in hepatocyte and podocytes and epithelial cells of the proximal tubule of the kidney 80, 81. The transcription factor promyelocytic leukaemia zinc finger protein (PLZF) is a significant component of the ZBTB16/PLZF-Cullin3-Roc1E3 ubiquitin ligase complex. Its expression shows a negative correlation with the key autophagy protein ATG14L, indicating that PLZF acts as a suppressor of autophagy 69, 78, 82, 83. P62/SQSTM1 is a commonly used marker protein in autophagic activity assays, and its expression level decreases during the autophagic process 84. Elevated hepatic antisense lncRNA AS-nnmt-PLZF was found to be accompanied by upregulation of hepatic triglyceride accumulation and its metabolism-related gene expression. In animal models of T2DM, hyperglycaemia has been shown to impair cellular autophagy, which is associated with upregulation of p62/SQSTM1 levels. Combined with previous findings, PLZF may be involved in the mechanism of DKD through the negative regulation of autophagy 85.

### Regulation of cellular pyroptosis

Cellular pyroptosis is a strongly pro-inflammatory process of programmed cell death that can play an important role in regulating lipid accumulation in the liver and kidney. Abnormal mitochondrial function during cell death reduces ATP synthesis, resulting in inadequate intracellular energy supply and subsequently promoting lipid synthesis. Additionally, cell pyroptosis decreases lipase activity, impeding intracellular lipolysis and contributing to lipid accumulation 86, 87. According to the different activation proteins, cellular pyroptosis is divided into the classical pathway cellular pyroptosis dependent on cysteine protease-1 (Caspase-1) and the non-classical pathway cellular pyroptosis dependent on Caspase-4/5/11, and the final execution protein of both pathways is GSDMD. NOD-like receptor protein 3 (NLRP3) is a multi-protein complex that shears caspase-1, which in turn mediates the shearing of GSDMD, exposing the GSDMD-N-terminus and forming a pore-membrane structure on the cell membrane, activating the classical pathway of cellular cellular death 88, 89. It has been demonstrated that inhibition of NLRP3 inflammatory vesicles promotes lipid accumulation in podocytes, which ameliorates DKD damage 90. Caspase-11 knockdown reduced hepatic steatosis and ballooning, whereas overexpression exacerbated the accumulation of lipids 91. Excess lipid accumulation produces lipid metabolites such as cholesterol crystals and free fatty acids, which can act as danger signals, activating NLRP3 inflammatory vesicles and inducing pyroptosis in macrophages, hepatocytes and hepatic stellate cells 92.

### Intestinal flora

Studies have revealed differences in fecal microbiota composition between patients with NAFLD or DKD and healthy subjects 93-96. Research involving germ-free mice, antibiotic interventions, and fecal microbiota transplantation has highlighted the essential role of the intestinal microbiota in influencing host lipid metabolism 97-99. The impact of gut microbiota on host lipid metabolism may be mediated through various metabolites produced by the gut microbiota, such as short-chain fatty acids (SCFAs), secondary bile acids, and trimethylamine (TMA)/trimethylamine oxide (TMAO), as well as pro-inflammatory bacterial-derived factors like lipopolysaccharides 98.

#### SCFAs

The intestinal flora can ferment undigested carbohydrates to produce SCFAs. Insufficient production of SCFAs has been observed in patients with DKD 93. SCFAs have a crucial effect on host metabolism. SCFAs not only serve as substrates for energy production, adipogenesis, gluconeogenesis and cholesterol synthesis 100, 101, but also as signalling molecules for the regulation of inflammation, oxidative stress and fibrosis by binding to specific G receptors associated with the GPR43 and GPR41. It has been shown that GPR43 prevents dietary induced obesity in mice 102. Activation of GPR43 on L cells increases the secretion of glucagon-like peptide-1, which in turn promotes insulin secretion 102, 103. SCFAs activate GPR43 in white adipose tissue, induce antilipolytic activity and improve glucose and lipid metabolism 104, 105. SCFAs control satiety and intestinal transshipment by activating GPR41 to increase peptide YY production 106. SCFAs have also been shown to activate PPARγ to increase energy expenditure to regulate lipid metabolism, reduce body weight and reduce hepatic triglyceride accumulation 107-109. Additionally, SCFAs can also reduce serum TG and TC levels by inhibiting fatty acid synthase activity in the liver 110.

#### Bile acids

Primary bile acids and secondary bile acids produced by the gut microbiota contribute to the maintenance of glucose lipid metabolic homeostasis in the liver and kidney. Clinical studies have shown that dysregulation of bile acid homeostasis and its associated signaling pathways is related to the development of NAFLD and DKD 111. NASH patients have increased concentrations of bile acids in serum and urine samples compared to healthy subjects 112, 113. In addition, plasma levels of glycine cholate, taurocholate, and glycine deoxycholate were increased in NASH patients compared to NAFLD patients 114. And the levels of glycine cholate and taurocholate correlated with the severity of portal inflammation, lobular inflammation, steatosis, and hepatocyte ballooning-like lesions, respectively 111, 115, 116. Similarly, changes in bile acid levels were found in the disease progression of DKD 117: a gradual increase in bile acid levels was observed in clinical patients when progressing from nondiabetes to diabetes, but a gradual decrease in diabetic patients when progressing to DKD 118. Analysing this state may be similar to the mechanism of decompensation. In diabetes, bile acid levels may initially increase. However, as DKD advances, this compensatory mechanism diminishes, exacerbating renal damage. Notably, DKD patients typically have lower bile acid levels than those without DKD, suggesting a potential kidney-protective role for bile acids. Ursodeoxycholic acid has been demonstrated to alleviate kidney injury in DKD by preventing podocyte apoptosis induced by endoplasmic reticulum stress 119.

In analysing the relationship between bile acids and liver and kidney, intestinal flora is essential for the conversion of bile acids. A new point of view revealed that microbial-derived previously uncharacterized 3-succinylcholic acid (3-sucCA) is inversely associated with liver damage in patients diagnosed with metabolic dysfunction-associated fatty liver disease 120. Specific-pathogen-free mice supplemented with 3-sucCA supplemented with 8 weeks of choline-deficient amino-acid-defined and high-fat diet reduced hepatic triglyceride levels, reduced hepatic inflammation and fibrosis, and reduced the progression of NAFLD 120, 121. Intestinal flora has an important effect on the progression of NAFLD and DKD by synthesising secondary bile acids and regulating the balance of glucose and lipid metabolism through farnesol X receptor (FXR), and G-protein coupled receptor (TGR5) 122-125. Supplementation of high-fat or db/db mice with either TGR5 or FXR agonists inhibits lipogenesis, induces energy expenditure and reduces hepatic and renal inflammation to reduce the development of NAFLD and DKD 124.

#### TMA/TMAO

Gut microbes possess the ability to metabolize nutrients such as choline and betaine into TMA. TMA can be further oxidized by flavin-containing monooxygenase 3 (FMO3) in the liver or gut microbes to TMAO 126-129. TMAO is excreted from the body through the kidneys 130. In instances of dysbiosis within the intestinal flora, levels of TMAO are elevated 131. Elevated TMAO levels trigger an augmented inflammatory response, which, in turn, exacerbates renal impairment and accelerates the progression of DKD 132. There is a suggestion that intra-host TMAO levels could be linked with NAFLD and DKD by mediating insulin resistance 133. Additionally, it has been proposed that TMAO might influence NAFLD by altering bile acid metabolism 134. Research has indicated that TMAO could induce insulin resistance by activating the hepatic PERK signaling pathway, consequently leading to disturbances in lipid metabolism 135. This intricate mechanism could potentially elucidate how TMAO contributes to the development of NAFLD.

#### LPS

**Figure 3:**
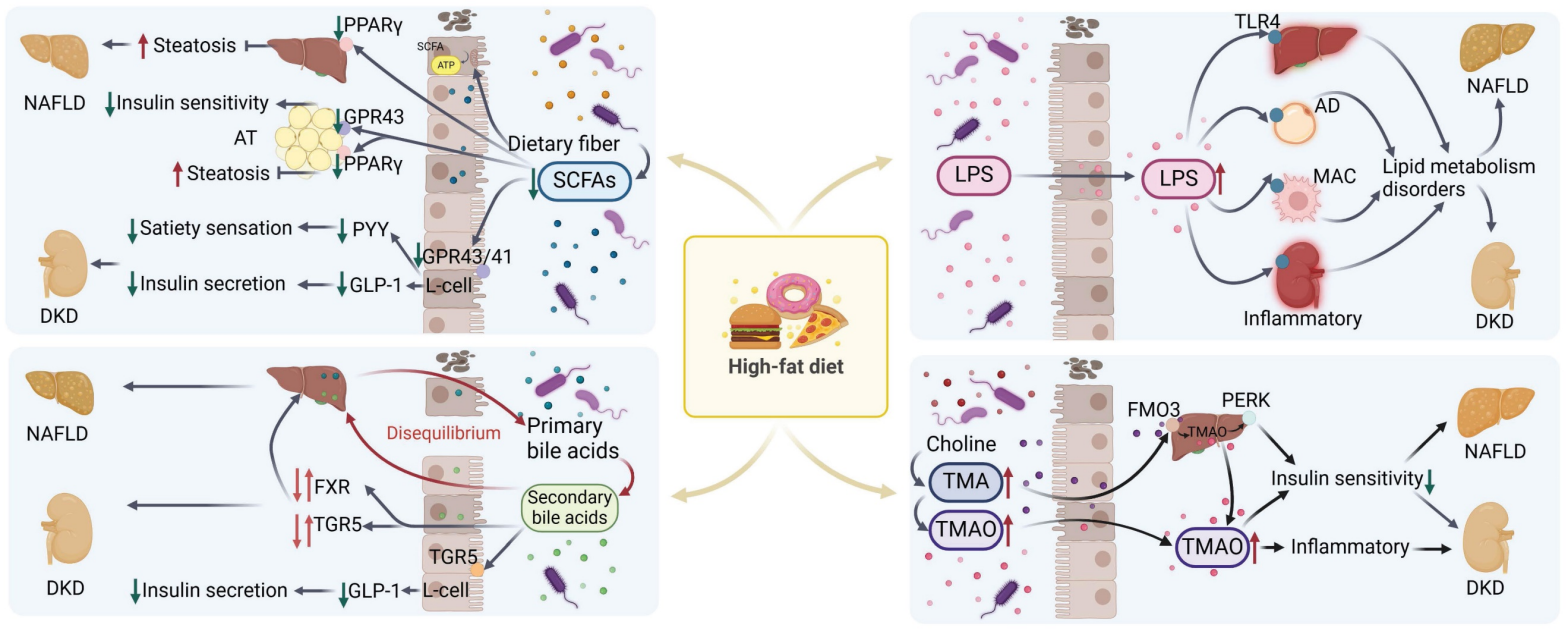
Mechanisms related to the effects of metabolites from disordered microbiota with NAFLD and DKD in high-fat diet. In the high-fat state, intestinal microbiota disorders are accompanied by a decrease in SCFAs, an imbalance in bile acid homeostasis, an increase in TMA/TMAO levels, and LPS translocation. These changes lead to dysregulation of related signaling pathways and metabolic disorders, which in turn lead to or exacerbate the progression of NAFLD and DKD. SCFAs: short chain fatty acids; AT: adipose tissue; AD: adipocyte; MAC: macrophage; TMA/TMAO: trimethylamine/trimetlylamine oxide; TLR4: toll-like receptor 4; NAFLD: non-alcoholic fatty liver disease; DKD: diabetic kidney disease. Red arrows up and down: Dysregulation of signaling pathways.

Lipopolysaccharide (LPS), or endotoxin, is a component of Gram-negative bacteria's outer membrane. It triggers inflammation by activating Toll-like receptor 4 (TLR4), found on immune cells like macrophages, as well as on hepatic and renal cells 136. A diet high in fat, coupled with increased exposure to fatty acids 137, has the potential to disrupt the integrity of the intestinal barrier 137, thereby facilitating the translocation of LPS 138, 139. Consequently, this leads to an elevation in the bloodstream levels of LPS, precipitating disorders associated with lipid metabolism, such as dyslipidemia, insulin resistance, and NAFLD 140. The translocation of LPS into the bloodstream can also result in its transportation to the kidney, where it induces renal impairment and accelerates the deposition of lipids within the kidneys, contributing to the progression of DKD 141, 142. The effects of various metabolites produced by the gut microbiota on lipid metabolism are shown in Figure 3. And table 1 summarises the similarities and differences in lipid deposition and metabolism between NAFLD and DKD described above.

## Treatment

### Western medicine

Researchers have long studied lipid-lowering therapies for NAFLD and DKD arising from metabolic syndrome's lipid metabolism disorders. Currently, lipid-lowering drugs primarily include TG-lowering agents like fibrates (e.g., fenofibrate), high-purity ω-3 unsaturated fatty acids, and TC-lowering medications such as cholesterol uptake inhibitors (e.g., ezetimibe) and statins. Additionally, glucose-lowering drugs like SGLT-2 inhibitors (e.g., riflouxate) and metformin are widely utilized in the clinical treatment of NAFLD and DKD, these medications target lipid and glucose levels to reduce lipid accumulation in hepatocytes and podocyte 143-148. Although statins are recommended, there is no evidence suggesting they slow NAFLD and DKD progression, often necessitating clinical combination therapy.

In addition to the therapeutic effects of medications, there are also adverse reactions that need to be addressed. Adverse muscle effects associated with statins include myalgia, myositis, myopathy, and rhabdomyolysis 149, 150. Long-term use of statins carries an increased risk of new-onset diabetes mellitus, known as the statin effect. When high-intensity statins are used, the incidence of new-onset diabetes is higher compared to moderate-intensity statins (9% versus 12%) 151. Currently, clinical administration through dose control effectively mitigates the renal toxicity of statins. A meta-analysis indicates that statin medications have no adverse effects on kidney function 152. Compared to patients taking low-potency statins, those taking high-potency statins have a 34% higher risk of hospitalization due to acute kidney injury (AKI) within 120 days of starting treatment 153. Additionally, a meta-analysis of 57 randomized controlled trials (RCTs) involving nearly 140,000 patients treated with statins for at least six months found that estimated glomerular filtration rate (eGFR) declined by 0.41 mL/min per 1.73 m² annually 154. It's important to note that according to these RCT results, statin use did not show a significant association with the development of end-stage kidney disease 154, 155. Regarding the hepatotoxicity of statins, a preliminary clinical trial observed elevated transaminase levels in approximately 2% of patients. A common side effect is asymptomatic elevation of liver enzyme activity, which typically resolves upon dose reduction 156. Adverse reactions to the cholesterol inhibitor ezetimibe are mild and transient, mainly presenting as headaches and gastrointestinal symptoms 157. The common adverse effects of the lipid-lowering drug fenofibrate resemble those of statins, including liver, muscle, and renal toxicity 158, 159.

New pathways and drugs targeting cellular lipid synthesis, uptake, transport, and metabolism have emerged. Liver and kidney, expressing high levels of FXR and LXR, are crucial in bile acid and cholesterol regulation 160. FXR can promote cholesterol metabolic conversion by negatively regulating cholesterol 7α-hydroxylase, as well as inducing expression of small heterodimeric chaperones to inhibit SREBP-1c transcription, which in turn down-regulates the activity of key enzymes of lipogenesis to restore normal lipid metabolism in NAFLD and DKD 161. Currently, obeticholic acid, a well-established and potent FXR agonist, was approved for marketing by the US FDA in 2016. An inverse agonist of LXR, SR9238, showed high potency against both LXRα and LXRβ, and was effective in inhibiting plasma cholesterol levels in a NAFLD mouse model that reduces hepatic lipogenesis and accumulation 162. The LXR agonist T0901317, on the other hand, stimulates LXRa expression in renal tubules and pumps lipids out of the cell by promoting ABCA1 transcription to reduce lipid accumulation and ameliorate inflammation and fibrosis. These common drugs are partly summarized in Table 2.

### Chinese Medicine Treatment

Traditional Chinese medicines (TCMs) and their compounds have a longstanding history and widespread popularity in China and parts of Asia, owing to their extensive theoretical foundation and minimal adverse effects. Recent studies involving animal models and clinical trials have substantiated the biological activities and therapeutic effectiveness of various TCM formulations in addressing metabolic syndrome 163-165.

Tangshen Formula (TSF) is a traditional Chinese medicine compound developed on the principles of "liver and kidney therapy." Pharmacokinetic research in rat plasma has revealed the presence of 44 detectable components from TSF 163, 166. Studies have demonstrated TSF's ability to mitigate lipid accumulation in the liver and kidney, ameliorate hepatic steatosis, and attenuate kidney damage in mice subjected to high-fat diets and db/db mice 167, 168. Differently, TSF intervention in NAFLD relies on the SIRT1-AMPK pathway, which mediates lipid autophagy 85, 169. In response to lipid deposition in DKD, TSF enhances lipid phagocytosis by regulating PLZF expression and modulating signalling pathways such as PGC- 1α/PPARα and PGC-1α/LXR/ABCA1. These effects help regulate renal lipid oxidation, promote renal cholesterol efflux, and ultimately reduce renal lipid deposition 168, 170. Gut microbiota disorders are closely related to metabolic diseases and may lead to increased absorption of endotoxins and other harmful substances into the bloodstream. TSF regulates the intestinal flora based on the "gut-kidney" and "gut-liver" axes, thereby reducing lipopolysaccharide levels and significantly improving inflammation and lipid deposition in NAFLD and DKD 99, 171-174.

Pien Tze Huang as a traditional Chinese herbal formula widely used in China and Southeast Asia 175. Recent research indicates that Pien Tze Huang can significantly improve methionine- and choline-deficient diet-induced steatosis and liver damage in mice. It achieves this by modulating gut microbiota, restoring intestinal barrier function, and modifying intestinal metabolites through the gut-liver axis, thus mitigating the progression of NASH 176. Moreover, research on diabetic rats indicates that oral administration of Pien Tze Huang can suppress inflammation, enhance energy production, and promote wound healing in diabetic patients 177.

In addition to herbal compounds, herbal monomers are crucial for improving lipid aggregation in NAFLD and DKD. Quercetin, a prominent flavonoid in TCM, has potent antioxidant properties and reduces lipid accumulation and the expression of SREBP-1 and XBP-1 in adipocytes and their adipogenic gene targets, thus linking closely to metabolic diseases related to glucolipid metabolism disorders 178-181. Berberine, an isoquinoline alkaloid in Chinese herbs, down-regulates LXR and SREBP1c, inhibits fatty acid synthesis, activates PGC-1α signaling, promotes mitochondrial energy homeostasis, and enhances fatty acid oxidation, thereby regulating lipid accumulation in hepatocytes, podocyte and proximal renal tubular cells 182-184. In addition, resveratrol, as a recognised Sirt1 agonist, can partially regulate the expression of SREBP-1 and ChREBP in the JAML/Sirt lipid synthesis pathway, and reduce high-fat diet-induced lipid deposition and lipotoxicity damage in the kidney of NAFLD and DKD mice 185-187. Table 2 summarises the common drugs and their associated mechanisms of action for the treatment of MetS lipid metabolism disorders liver and kidney injury.

## Conclusion and Prospects

Extensive clinical and basic research confirms a close correlation between Mets, NAFLD and DKD 12, highlighting their roles in lipid metabolism disorders and underscoring the importance of lipid metabolism. While there is considerable literature on the pathogenesis of each condition, the complexity of organ crosstalk and its underlying mechanisms remain areas with significant research potential. Future studies should place greater emphasis on the interactions between organs.

In this article, we have not only reviewed the effectiveness of Western medicine in treating the diseases discussed but also explored the potential of traditional Chinese medicine. Traditional Chinese medicine can serve as a complementary or alternative treatment to Western medicine. However, its efficacy currently relies primarily on clinical experience and small clinical studies. To substantiate these treatment methods, there is a pressing need for large-scale randomized clinical trials and comprehensive mechanistic research. Employing modern scientific techniques and conducting detailed pharmacological studies are essential to assess their safety and effectiveness.

## Figures and Tables

**Table 1:** Differences and similarities between NAFLD and DKD in lipid deposition and metabolism.

Lipid metabolism		Commonality	Differences			
		Lipid regulatory proteins/metabolites			Cell types	
			NAFLD	DKD	NAFLD	DKD
Lipid metabolic processes	Circulating lipid uptake	FATP2, FATP4, CD36, A-FABP, L-FABP	FATP5, E-FABP		Hepatocytes	Proximal renal tubular cells
	Lipid synthesis and deposition	SREBP1c, ChREBP			Hepatocytes	Podocytes
	Fatty acid oxidation	PPARα, PGC-1α, CPT1, Peroxisome	ACOX1	Succinate	Hepatocytes	Proximal renal tubular cells, Podocytes
	Cholesterol efflux	ABCA1, LXRα			Hepatocytes	Proximal renal tubular cells
Pathway of lipid metabolism	Regulation of oxidative stress	Nrf2			Hepatocytes	Proximal renal tubular cells
	Programmed Cell Death	Autophagy: JAK/STAT, SIRT1, AMPK, mTOR, PLZF Pyroptosis: Caspase	Caspase-11	Caspase-1	Hepatocytes, Hepatic stellate cells	Proximal renal tubular cells, Podocytes
	Intestinal flora	SCFAs: GPR43, GPR41, PPARγ Bile acids: FXR, TGR5, TMA/TMAO LPS: TLR4	Glycine cholate, Taurocholate, and Glycine deoxycholate	Ursodeoxycholic acid		

**Table 2:** The common drugs and their associated mechanisms of action for the treatment of MetS lipid metabolism disorders liver and kidney injury

Medication	Classification	Name of drug	Model	Targeted molecular	Pathway	Ref
Western medicine	Lipid-lowering drugs	Statins (Atorvastatin)	renal tubular epithelial cell, APOE*3-Leiden mice	HMG-CoA, PPARα		143
		Fibrates (Fenofibrate)	db/db mice,renal tubular epithelial cel	TFEB, MCAD	AMPK/FOXA2	144
		cholesterol absorption inhibitors (Ezetimibe)	db/db mice	lipocalin receptor 1		145
	Glucose-lowering drugs	SGLT-2 inhibitors (Empagliflozin)	C57BL/6J mice, HepG2 cell		AMPK/mTOR, AGEs-RAGE	146
		Metformin	ob/ob mouse	SIRT1		143
			Sprague-Dawley rats	AMPK, ACC		188
	Immunosuppressive agents	mTORC1 inhibitor (Rapamycin)	Flcn^lox/lox^ mice	TFE3,		145
			human renal proximal, tubular epithelial cells	SREBP1, ADRP		
	Novel targeted inhibitors	FXR agonists	Wistar rats	FXR, SREBP-1		161
		LXR agonists	C57 Bl/6 mice,murine proximal tubule cells	LXRα, ABCA1 t		189
		LXR inverse agonists	C57BL6 DIO mice, HepG2 cell	LXRα, LXRβ		162
Traditional Chinese medicine	Traditional Chinese medicine compound	TSF	Wistar rats	PLZF	PGC-1α/P PARα, PGC-1α/LXR/ABCA1	170
			C57BL/6 J mice, HepG2 cell	AMPK, TFEB	mTOR-TFEBs	85
			db/db mice	Sirt1, AMPK, PPARα, MLYCD	SIRT1-AMPK	167, 169
			Wistar rats	TLR4		
	Traditional Chinese medicine monomers	Berberine	ob/ob mice	AMPK, pAMKP, PGC-1α, CPT1, pACC, ACC, CD36	AMPK/PGC-1α	190
			db/db mice	AMPK, ACC, p-ACC, CPT-1	AMPK	191
			Wistar rats	IR, IRS-2		192
			db/db mice	COXIII, COXIV, CPT2, NRF1, PGC-1α, UCP1, UCP2, IgG, p-AMPK, p-ACC	AMPK/PGC-1α	191
			ACC 1/2 whole body KI mice	P-ACC, ACC		193
		Resveratrol	Wistar rats	Rβ, 30 IRS-1, IRS-2, eNOS, PI3K, Akt, FOXO3a	IRS-1, IRS-2, PI3K, Akt, mTOR	192
			Wistar rats	Slc2a2, GLUT2, Pckl, G6pc, Pparγ, RBP4, Glut308, p-AKT, IRS-1		194
		Quercetin	Sprague-Dawley rats	MDA, GSH/GSSG, CAT, GSH-Px, SOD, α-SMA, TGF-β	TGF-b2/PI3K/Akt	195
			C57BL/6 J mice	TC, LDL, HDL, TG, ALT, AST, TNF-α, OPN, SOCS3, iNOS	PI3K/Akt	196
			HepG2 cell		ACACA/AMPK/PP2A	179
			Leprdb/Leprdb (db/db) mice	LDLr, HMGCR, SPEBP-2, SCAP	SCAP-SREBP2-LDLr	180

## Abbreviations

MetS: Metabolic syndrome
NAFLD: non-alcoholic fatty liver disease
DKD: diabetic kidney disease
IR: insulin resistance
CM: chylomicrons
VLDL: very low-density lipoproteins
NASH: non-alcoholic steatohepatitis
CKD: chronic kidney disease
FATP: fatty acid transport protein
CD36: cluster of differentiation 36
FABP: fatty acid-binding proteins
Ox-LDL: oxidised low-density lipoproteins
ACLY: ATP citrate lyase
SREBP1c: sterol regulatory element-binding protein 1c
ChREBP: carbohydrate response element binding protein
ACC: acetyl Coenzyme A carboxylase
FASN: fatty acid synthase
HIF-1α: hypoxia-inducible factor-1α
HFrD: high fructose diet
PPARα: peroxisome proliferator-activated receptor α
PGC-1α: peroxisome proliferator-activated receptor-gamma coactivator-1alpha
CPT1: carnitine palmitoyltransferase 1
H2O2: hydrogen peroxide
ABCA1: ATP-binding cassette transporter protein A1
LXR: liver X receptor
ROS: reactive oxygen species
Nrf2: nuclear factor E2-related factor 2
AMPK: adenosine-phosphate-activated protein kinase
PLZF: promyelocytic leukaemia zinc finger protein
Caspase-1: cysteine protease-1
NLRP3: NOD-like receptor protein 3
SCFAs: short-chain fatty acids
TMA: trimethylamine
TMAO: trimethylamine oxide
3-sucCA: 3-succinylcholic acid
FXR: farnesol X receptor
FMO3: flavin-containing monooxygenase 3
LPS: lipopolysaccharide
TLR4: toll-like receptor 4
AKI: acute kidney injury
RCTs: randomized controlled trials
eGFR: estimated glomerular filtration rate
TCMs: traditional Chinese medicines
TSF: Tangshen Formula

## References

[1] Radu F, Potcovaru C-G, Salmen T, Filip PV, Pop C, Fierbințeanu-Braticievici C (2023). The Link between NAFLD and Metabolic Syndrome. Diagnostics.

[2] Fahed G, Aoun L, Bou Zerdan M, Allam S, Bou Zerdan M, Bouferraa Y (2022). Metabolic Syndrome: Updates on Pathophysiology and Management in 2021. International Journal of Molecular Sciences.

[3] Hirode G, Wong RJ (2020). Trends in the Prevalence of Metabolic Syndrome in the United States, 2011-2016. JAMA.

[4] Lu J, Wang L, Li M, Xu Y, Jiang Y, Wang W (2017). Metabolic Syndrome Among Adults in China: The 2010 China Noncommunicable Disease Surveillance. J Clin Endocrinol Metab.

[5] Gurka MJ, Golden SH, Musani SK, Sims M, Vishnu A, Guo Y (2017). Independent associations between a metabolic syndrome severity score and future diabetes by sex and race: the Atherosclerosis Risk In Communities Study and Jackson Heart Study. Diabetologia.

[6] Li S, Cui M, Liu Y, Liu X, Luo L, Zhao W (2024). Metabolic Profiles of Type 2 Diabetes and Their Association With Renal Complications. The Journal of Clinical Endocrinology & Metabolism.

[7] Duan Y, Zhang W, Li Z, Niu Y, Chen Y, Liu X (2022). Predictive ability of obesity- and lipid-related indicators for metabolic syndrome in relatively healthy Chinese adults. Front Endocrinol (Lausanne).

[8] Schoeler M, Caesar R (2019). Dietary lipids, gut microbiota and lipid metabolism. Rev Endocr Metab Disord.

[9] Hodson L, Gunn PJ (2019). The regulation of hepatic fatty acid synthesis and partitioning: the effect of nutritional state. Nat Rev Endocrinol.

[10] Chang J, Yan J, Li X, Liu N, Zheng R, Zhong Y (2021). Update on the Mechanisms of Tubular Cell Injury in Diabetic Kidney Disease. Front Med (Lausanne).

[11] DeBose-Boyd RA (2018). Significance and regulation of lipid metabolism. Semin Cell Dev Biol.

[12] Li X, Bhattacharya D, Yuan Y, Wei C, Zhong F, Ding F (2024). Chronic kidney disease in a murine model of non-alcoholic steatohepatitis (NASH). Kidney International.

[13] de Carvalho C, Caramujo MJ (2018). The Various Roles of Fatty Acids. Molecules.

[14] Falcon A, Doege H, Fluitt A, Tsang B, Watson N, Kay MA (2010). FATP2 is a hepatic fatty acid transporter and peroxisomal very long-chain acyl-CoA synthetase. Am J Physiol Endocrinol Metab.

[15] Doege H, Grimm D, Falcon A, Tsang B, Storm TA, Xu H (2008). Silencing of hepatic fatty acid transporter protein 5 *in vivo* reverses diet-induced non-alcoholic fatty liver disease and improves hyperglycemia. J Biol Chem.

[16] Zhu L, Baker SS, Liu W, Tao MH, Patel R, Nowak NJ (2011). Lipid in the livers of adolescents with nonalcoholic steatohepatitis: combined effects of pathways on steatosis. Metabolism.

[17] Westerbacka J, Kolak M, Kiviluoto T, Arkkila P, Siren J, Hamsten A (2007). Genes involved in fatty acid partitioning and binding, lipolysis, monocyte/macrophage recruitment, and inflammation are overexpressed in the human fatty liver of insulin-resistant subjects. Diabetes.

[18] Auinger A, Valenti L, Pfeuffer M, Helwig U, Herrmann J, Fracanzani AL (2010). A promoter polymorphism in the liver-specific fatty acid transport protein 5 is associated with features of the metabolic syndrome and steatosis. Horm Metab Res.

[19] Bloksgaard M, Neess D, Faergeman NJ, Mandrup S (2014). Acyl-CoA binding protein and epidermal barrier function. Biochim Biophys Acta.

[20] Khan S, Cabral PD, Schilling WP, Schmidt ZW, Uddin AN, Gingras A (2018). Kidney Proximal Tubule Lipoapoptosis Is Regulated by Fatty Acid Transporter-2 (FATP2). J Am Soc Nephrol.

[21] Khan S, Gaivin R, Abramovich C, Boylan M, Calles J, Schelling JR (2020). Fatty acid transport protein-2 regulates glycemic control and diabetic kidney disease progression. JCI Insight.

[22] Rada P, Gonzalez-Rodriguez A, Garcia-Monzon C, Valverde AM (2020). Understanding lipotoxicity in NAFLD pathogenesis: is CD36 a key driver?. Cell Death Dis.

[23] Wilson CG, Tran JL, Erion DM, Vera NB, Febbraio M, Weiss EJ (2016). Hepatocyte-Specific Disruption of CD36 Attenuates Fatty Liver and Improves Insulin Sensitivity in HFD-Fed Mice. Endocrinology.

[24] Ipsen DH, Lykkesfeldt J, Tveden-Nyborg P (2018). Molecular mechanisms of hepatic lipid accumulation in non-alcoholic fatty liver disease. Cell Mol Life Sci.

[25] Koonen DP, Jacobs RL, Febbraio M, Young ME, Soltys CL, Ong H (2007). Increased hepatic CD36 expression contributes to dyslipidemia associated with diet-induced obesity. Diabetes.

[26] Miquilena-Colina ME, Lima-Cabello E, Sanchez-Campos S, Garcia-Mediavilla MV, Fernandez-Bermejo M, Lozano-Rodriguez T (2011). Hepatic fatty acid translocase CD36 upregulation is associated with insulin resistance, hyperinsulinaemia and increased steatosis in non-alcoholic steatohepatitis and chronic hepatitis C. Gut.

[27] Hou Y, Tan E, Shi H, Ren X, Wan X, Wu W (2024). Mitochondrial oxidative damage reprograms lipid metabolism of renal tubular epithelial cells in the diabetic kidney. Cell Mol Life Sci.

[28] Kennedy DJ, Chen Y, Huang W, Viterna J, Liu J, Westfall K (2013). CD36 and Na/K-ATPase-alpha1 form a proinflammatory signaling loop in kidney. Hypertension.

[29] Feng L, Gu C, Li Y, Huang J (2017). High Glucose Promotes CD36 Expression by Upregulating Peroxisome Proliferator-Activated Receptor gamma Levels to Exacerbate Lipid Deposition in Renal Tubular Cells. Biomed Res Int.

[30] Owczarek A, Gieczewska KB, Jarzyna R, Frydzinska Z, Winiarska K (2021). Transcription Factor ChREBP Mediates High Glucose-Evoked Increase in HIF-1α Content in Epithelial Cells of Renal Proximal Tubules. International Journal of Molecular Sciences.

[31] Smathers RL, Galligan JJ, Shearn CT, Fritz KS, Mercer K, Ronis M (2013). Susceptibility of L-FABP-/- mice to oxidative stress in early-stage alcoholic liver. J Lipid Res.

[32] Jin R, Hao J, Yi Y, Sauter E, Li B (2021). Regulation of macrophage functions by FABP-mediated inflammatory and metabolic pathways. Biochim Biophys Acta Mol Cell Biol Lipids.

[33] Yan T, Luo Y, Yan N, Hamada K, Zhao N, Xia Y (2023). Intestinal peroxisome proliferator-activated receptor alpha-fatty acid-binding protein 1 axis modulates nonalcoholic steatohepatitis. Hepatology.

[34] Li X, Ma T-K, Wang M, Zhang X-D, Liu T-Y, Liu Y (2023). YY1-induced upregulation of LncRNA-ARAP1-AS2 and ARAP1 promotes diabetic kidney fibrosis via aberrant glycolysis associated with EGFR/PKM2/HIF-1α pathway. Frontiers in Pharmacology.

[35] Watt J, Kurth MJ, Reid CN, Lamont JV, Fitzgerald P, Ruddock MW (2022). Non-alcoholic fatty liver disease-A pilot study investigating early inflammatory and fibrotic biomarkers of NAFLD with alcoholic liver disease. Front Physiol.

[36] Charlton M, Viker K, Krishnan A, Sanderson S, Veldt B, Kaalsbeek AJ (2009). Differential expression of lumican and fatty acid binding protein-1: new insights into the histologic spectrum of nonalcoholic fatty liver disease. Hepatology.

[37] Watanabe S, Ichikawa D, Sugaya T, Ohata K, Inoue K, Hoshino S (2018). Urinary Level of Liver-Type Fatty Acid Binding Protein Reflects the Degree of Tubulointerstitial Damage in Polycystic Kidney Disease. Kidney Blood Press Res.

[38] Negishi K, Noiri E, Maeda R, Portilla D, Sugaya T, Fujita T (2008). Renal L-type fatty acid-binding protein mediates the bezafibrate reduction of cisplatin-induced acute kidney injury. Kidney Int.

[39] Chen S, Sun S, Feng Y, Li X, Yin G, Liang P (2023). Diosgenin attenuates nonalcoholic hepatic steatosis through the hepatic FXR-SHP-SREBP1C/PPARalpha/CD36 pathway. Eur J Pharmacol.

[40] Ju W, Greene CS, Eichinger F, Nair V, Hodgin JB, Bitzer M (2013). Defining cell-type specificity at the transcriptional level in human disease. Genome Res.

[41] Nakagawa S, Nishihara K, Miyata H, Shinke H, Tomita E, Kajiwara M (2015). Molecular Markers of Tubulointerstitial Fibrosis and Tubular Cell Damage in Patients with Chronic Kidney Disease. PLoS One.

[42] Huang TS, Wu T, Wu YD, Li XH, Tan J, Shen CH (2023). Long-term statins administration exacerbates diabetic nephropathy via ectopic fat deposition in diabetic mice. Nat Commun.

[43] Fu Y, Sun Y, Wang M, Hou Y, Huang W, Zhou D (2020). Elevation of JAML Promotes Diabetic Kidney Disease by Modulating Podocyte Lipid Metabolism. Cell Metab.

[44] Dentin R, Benhamed F, Hainault I, Fauveau V, Foufelle F, Dyck JR (2006). Liver-specific inhibition of ChREBP improves hepatic steatosis and insulin resistance in ob/ob mice. Diabetes.

[45] Zhang D, Tong X, VanDommelen K, Gupta N, Stamper K, Brady GF (2017). Lipogenic transcription factor ChREBP mediates fructose-induced metabolic adaptations to prevent hepatotoxicity. J Clin Invest.

[46] Kersten S, Stienstra R (2017). The role and regulation of the peroxisome proliferator activated receptor alpha in human liver. Biochimie.

[47] Nassir F, Ibdah JA (2014). Role of mitochondria in nonalcoholic fatty liver disease. Int J Mol Sci.

[48] Chen Z, Tian R, She Z, Cai J, Li H (2020). Role of oxidative stress in the pathogenesis of nonalcoholic fatty liver disease. Free Radic Biol Med.

[49] Tahri-Joutey M, Andreoletti P, Surapureddi S, Nasser B, Cherkaoui-Malki M, Latruffe N (2021). Mechanisms Mediating the Regulation of Peroxisomal Fatty Acid Beta-Oxidation by PPARalpha. Int J Mol Sci.

[50] Francque S, Verrijken A, Caron S, Prawitt J, Paumelle R, Derudas B (2015). PPARalpha gene expression correlates with severity and histological treatment response in patients with non-alcoholic steatohepatitis. J Hepatol.

[51] Videla LA, Pettinelli P (2012). Misregulation of PPAR Functioning and Its Pathogenic Consequences Associated with Nonalcoholic Fatty Liver Disease in Human Obesity. PPAR Res.

[52] Wu L, Liu C, Chang DY, Zhan R, Zhao M, Man Lam S (2021). The Attenuation of Diabetic Nephropathy by Annexin A1 via Regulation of Lipid Metabolism Through the AMPK/PPARalpha/CPT1b Pathway. Diabetes.

[53] Park MJ, Kim DI, Lim SK, Choi JH, Kim JC, Yoon KC (2014). Thioredoxin-interacting protein mediates hepatic lipogenesis and inflammation via PRMT1 and PGC-1alpha regulation *in vitro* and *in vivo*. J Hepatol.

[54] Xue H, Li P, Luo Y, Wu C, Liu Y, Qin X (2019). Salidroside stimulates the Sirt1/PGC-1alpha axis and ameliorates diabetic nephropathy in mice. Phytomedicine.

[55] Dixon ED, Nardo AD, Claudel T, Trauner M (2021). The Role of Lipid Sensing Nuclear Receptors (PPARs and LXR) and Metabolic Lipases in Obesity, Diabetes and NAFLD. Genes (Basel).

[56] Fang C, Pan J, Qu N, Lei Y, Han J, Zhang J (2022). The AMPK pathway in fatty liver disease. Front Physiol.

[57] Wang J, Zeng J, Yin G, Deng Z, Wang L, Liu J (2023). Long non-coding RNA FABP5P3/miR-22 axis improves TGFbeta1-induced fatty acid oxidation deregulation and fibrotic changes in proximal tubular epithelial cells of renal fibrosis. Cell Cycle.

[58] Park HS, Song JW, Park JH, Lim BK, Moon OS, Son HY (2021). TXNIP/VDUP1 attenuates steatohepatitis via autophagy and fatty acid oxidation. Autophagy.

[59] Chen J, Chen J, Fu H, Li Y, Wang L, Luo S (2019). Hypoxia exacerbates nonalcoholic fatty liver disease via the HIF-2alpha/PPARalpha pathway. Am J Physiol Endocrinol Metab.

[60] Tserga A, Pouloudi D, Saulnier-Blache JS, Stroggilos R, Theochari I, Gakiopoulou H (2022). Proteomic Analysis of Mouse Kidney Tissue Associates Peroxisomal Dysfunction with Early Diabetic Kidney Disease. Biomedicines.

[61] Chen X, Chen S, Pang J, Huang R, You Y, Zhang H (2023). Hepatic steatosis aggravates atherosclerosis via small extracellular vesicle-mediated inhibition of cellular cholesterol efflux. J Hepatol.

[62] Phillips MC (2018). Is ABCA1 a lipid transfer protein?. J Lipid Res.

[63] Ghanem SE, Elsabaawy MM, Abdelkareem MM, Helal ML, Othman W, Elsayed M (2023). Evaluation of ABCA1 gene polymorphism as a prognostic index of fibrosis progression in NAFLD patients. Endocrinology, Diabetes & Metabolism.

[64] Zhang J, Wu Y, Zhang J, Zhang R, Wang Y, Liu F (2023). ABCA1 deficiency-mediated glomerular cholesterol accumulation exacerbates glomerular endothelial injury and dysfunction in diabetic kidney disease. Metabolism.

[65] Zelcer N, Hong C, Boyadjian R, Tontonoz P (2009). LXR regulates cholesterol uptake through Idol-dependent ubiquitination of the LDL receptor. Science.

[66] Fan L, Lai R, Ma N, Dong Y, Li Y, Wu Q (2021). miR-552-3p modulates transcriptional activities of FXR and LXR to ameliorate hepatic glycolipid metabolism disorder. J Hepatol.

[67] Wojcik P, Gegotek A, Zarkovic N, Skrzydlewska E (2021). Oxidative Stress and Lipid Mediators Modulate Immune Cell Functions in Autoimmune Diseases. Int J Mol Sci.

[68] Li X, Xu B, Wu J, Pu Y, Wan S, Zeng Y (2022). Maresin 1 Alleviates Diabetic Kidney Disease via LGR6-Mediated cAMP-SOD2-ROS Pathway. Oxidative Medicine and Cellular Longevity.

[69] Yang X, Jin Z, Lin D, Shen T, Zhang J, Li D (2022). FGF21 alleviates acute liver injury by inducing the SIRT1-autophagy signalling pathway. J Cell Mol Med.

[70] Yao Y, Luo ZP, Li HW, Wang SX, Wu YC, Hu Y (2023). P38gamma modulates the lipid metabolism in non-alcoholic fatty liver disease by regulating the JAK-STAT signaling pathway. FASEB J.

[71] Opazo-Rios L, Sanchez Matus Y, Rodrigues-Diez RR, Carpio D, Droguett A, Egido J (2020). Anti-inflammatory, antioxidant and renoprotective effects of SOCS1 mimetic peptide in the BTBR ob/ob mouse model of type 2 diabetes. BMJ Open Diabetes Res Care.

[72] Nguyen TB, Olzmann JA (2017). Lipid droplets and lipotoxicity during autophagy. Autophagy.

[73] Gluchowski NL, Becuwe M, Walther TC, Farese RV Jr (2017). Lipid droplets and liver disease: from basic biology to clinical implications. Nat Rev Gastroenterol Hepatol.

[74] Kashima J, Shintani-Ishida K, Nakajima M, Maeda H, Unuma K, Uchiyama Y (2014). Immunohistochemical study of the autophagy marker microtubule-associated protein 1 light chain 3 in normal and steatotic human livers. Hepatol Res.

[75] Carotti S, Aquilano K, Zalfa F, Ruggiero S, Valentini F, Zingariello M (2020). Lipophagy Impairment Is Associated With Disease Progression in NAFLD. Front Physiol.

[76] Korovila I, Hohn A, Jung T, Grune T, Ott C (2021). Reduced Liver Autophagy in High-Fat Diet Induced Liver Steatosis in New Zealand Obese Mice. Antioxidants (Basel).

[77] Han Y, Xiong S, Zhao H, Yang S, Yang M, Zhu X (2021). Lipophagy deficiency exacerbates ectopic lipid accumulation and tubular cells injury in diabetic nephropathy. Cell Death Dis.

[78] Yacoub R, Lee K, He JC (2014). The Role of SIRT1 in Diabetic Kidney Disease. Front Endocrinol (Lausanne).

[79] Chen D, Liu Y, Chen J, Lin H, Guo H, Wu Y (2021). JAK/STAT pathway promotes the progression of diabetic kidney disease via autophagy in podocytes. Eur J Pharmacol.

[80] Xu C, Wang L, Fozouni P, Evjen G, Chandra V, Jiang J (2020). SIRT1 is downregulated by autophagy in senescence and ageing. Nat Cell Biol.

[81] Huynh C, Ryu J, Lee J, Inoki A, Inoki K (2022). Nutrient-sensing mTORC1 and AMPK pathways in chronic kidney diseases. Nature Reviews Nephrology.

[82] Yan LS, Zhang SF, Luo G, Cheng BC, Zhang C, Wang YW (2022). Schisandrin B mitigates hepatic steatosis and promotes fatty acid oxidation by inducing autophagy through AMPK/mTOR signaling pathway. Metabolism.

[83] Yin H, Zuo Z, Yang Z, Guo H, Fang J, Cui H (2021). Nickel induces autophagy via PI3K/AKT/mTOR and AMPK pathways in mouse kidney. Ecotoxicol Environ Saf.

[84] Kim YC, Guan KL (2015). mTOR: a pharmacologic target for autophagy regulation. J Clin Invest.

[85] Wang Y, Zhao H, Li X, Wang Q, Yan M, Zhang H (2019). Formononetin alleviates hepatic steatosis by facilitating TFEB-mediated lysosome biogenesis and lipophagy. J Nutr Biochem.

[86] Lynch L, Michelet X, Zhang S, Brennan PJ, Moseman A, Lester C (2015). Regulatory iNKT cells lack expression of the transcription factor PLZF and control the homeostasis of T(reg) cells and macrophages in adipose tissue. Nat Immunol.

[87] Cheng ZY, He TT, Gao XM, Zhao Y, Wang J (2021). ZBTB Transcription Factors: Key Regulators of the Development, Differentiation and Effector Function of T Cells. Front Immunol.

[88] Qiu YY, Tang LQ (2016). Roles of the NLRP3 inflammasome in the pathogenesis of diabetic nephropathy. Pharmacol Res.

[89] Hutton HL, Ooi JD, Holdsworth SR, Kitching AR (2016). The NLRP3 inflammasome in kidney disease and autoimmunity. Nephrology (Carlton).

[90] Wu M, Yang Z, Zhang C, Shi Y, Han W, Song S (2021). Inhibition of NLRP3 inflammasome ameliorates podocyte damage by suppressing lipid accumulation in diabetic nephropathy. Metabolism.

[91] Drummer Ct, Saaoud F, Jhala NC, Cueto R, Sun Y, Xu K (2023). Caspase-11 promotes high-fat diet-induced NAFLD by increasing glycolysis, OXPHOS, and pyroptosis in macrophages. Front Immunol.

[92] Farrell GC, Haczeyni F, Chitturi S (2018). Pathogenesis of NASH: How Metabolic Complications of Overnutrition Favour Lipotoxicity and Pro-Inflammatory Fatty Liver Disease. Adv Exp Med Biol.

[93] Al Khodor S, Shatat IF (2017). Gut microbiome and kidney disease: a bidirectional relationship. Pediatr Nephrol.

[94] Da Silva HE, Teterina A, Comelli EM, Taibi A, Arendt BM, Fischer SE (2018). Nonalcoholic fatty liver disease is associated with dysbiosis independent of body mass index and insulin resistance. Sci Rep.

[95] Michail S, Lin M, Frey MR, Fanter R, Paliy O, Hilbush B (2015). Altered gut microbial energy and metabolism in children with non-alcoholic fatty liver disease. FEMS Microbiol Ecol.

[96] Del Chierico F, Nobili V, Vernocchi P, Russo A, De Stefanis C, Gnani D (2017). Gut microbiota profiling of pediatric nonalcoholic fatty liver disease and obese patients unveiled by an integrated meta-omics-based approach. Hepatology.

[97] Backhed F, Ding H, Wang T, Hooper LV, Koh GY, Nagy A (2004). The gut microbiota as an environmental factor that regulates fat storage. Proc Natl Acad Sci U S A.

[98] Velagapudi VR, Hezaveh R, Reigstad CS, Gopalacharyulu P, Yetukuri L, Islam S (2010). The gut microbiota modulates host energy and lipid metabolism in mice. J Lipid Res.

[99] Wang S, Li X, Zhang B, Li Y, Chen K, Qi H (2024). Tangshen formula targets the gut microbiota to treat non-alcoholic fatty liver disease in HFD mice: A 16S rRNA and non-targeted metabolomics analyses. Biomed Pharmacother.

[100] Bergman EN (1990). Energy contributions of volatile fatty acids from the gastrointestinal tract in various species. Physiol Rev.

[101] den Besten G, Lange K, Havinga R, van Dijk TH, Gerding A, van Eunen K (2013). Gut-derived short-chain fatty acids are vividly assimilated into host carbohydrates and lipids. Am J Physiol Gastrointest Liver Physiol.

[102] Magliocca G, Mone P, Di Iorio BR, Heidland A, Marzocco S (2022). Short-Chain Fatty Acids in Chronic Kidney Disease: Focus on Inflammation and Oxidative Stress Regulation. Int J Mol Sci.

[103] Chambers ES, Viardot A, Psichas A, Morrison DJ, Murphy KG, Zac-Varghese SE (2015). Effects of targeted delivery of propionate to the human colon on appetite regulation, body weight maintenance and adiposity in overweight adults. Gut.

[104] Kimura I, Ozawa K, Inoue D, Imamura T, Kimura K, Maeda T (2013). The gut microbiota suppresses insulin-mediated fat accumulation via the short-chain fatty acid receptor GPR43. Nat Commun.

[105] Robertson MD, Bickerton AS, Dennis AL, Vidal H, Frayn KN (2005). Insulin-sensitizing effects of dietary resistant starch and effects on skeletal muscle and adipose tissue metabolism. Am J Clin Nutr.

[106] Samuel BS, Shaito A, Motoike T, Rey FE, Backhed F, Manchester JK (2008). Effects of the gut microbiota on host adiposity are modulated by the short-chain fatty-acid binding G protein-coupled receptor, Gpr41. Proc Natl Acad Sci U S A.

[107] den Besten G, Bleeker A, Gerding A, van Eunen K, Havinga R, van Dijk TH (2015). Short-Chain Fatty Acids Protect Against High-Fat Diet-Induced Obesity via a PPARgamma-Dependent Switch From Lipogenesis to Fat Oxidation. Diabetes.

[108] Gao Z, Yin J, Zhang J, Ward RE, Martin RJ, Lefevre M (2009). Butyrate improves insulin sensitivity and increases energy expenditure in mice. Diabetes.

[109] Alex S, Lange K, Amolo T, Grinstead JS, Haakonsson AK, Szalowska E (2013). Short-chain fatty acids stimulate angiopoietin-like 4 synthesis in human colon adenocarcinoma cells by activating peroxisome proliferator-activated receptor gamma. Mol Cell Biol.

[110] Larkin TA, Astheimer LB, Price WE (2009). Dietary combination of soy with a probiotic or prebiotic food significantly reduces total and LDL cholesterol in mildly hypercholesterolaemic subjects. Eur J Clin Nutr.

[111] Chu H, Duan Y, Yang L, Schnabl B (2019). Small metabolites, possible big changes: a microbiota-centered view of non-alcoholic fatty liver disease. Gut.

[112] Clifford BL, Sedgeman LR, Williams KJ, Morand P, Cheng A, Jarrett KE (2021). FXR activation protects against NAFLD via bile-acid-dependent reductions in lipid absorption. Cell Metab.

[113] Wagner M, Halilbasic E, Marschall HU, Zollner G, Fickert P, Langner C (2005). CAR and PXR agonists stimulate hepatic bile acid and bilirubin detoxification and elimination pathways in mice. Hepatology.

[114] Smirnova E, Muthiah MD, Narayan N, Siddiqui MS, Puri P, Luketic VA (2022). Metabolic reprogramming of the intestinal microbiome with functional bile acid changes underlie the development of NAFLD. Hepatology.

[115] Zhang X, Coker OO, Chu ES, Fu K, Lau HCH, Wang YX (2021). Dietary cholesterol drives fatty liver-associated liver cancer by modulating gut microbiota and metabolites. Gut.

[116] Aragones G, Colom-Pellicer M, Aguilar C, Guiu-Jurado E, Martinez S, Sabench F (2020). Circulating microbiota-derived metabolites: a "liquid biopsy?. Int J Obes (Lond).

[117] Pazzi P, Morsiani E, Vilei MT, Granato A, Rozga J, Demetriou AA (2002). Serum bile acids in patients with liver failure supported with a bioartificial liver. Aliment Pharmacol Ther.

[118] Xiao X, Zhang J, Ji S, Qin C, Wu Y, Zou Y (2022). Lower bile acids as an independent risk factor for renal outcomes in patients with type 2 diabetes mellitus and biopsy-proven diabetic kidney disease. Front Endocrinol (Lausanne).

[119] Cao AL, Wang L, Chen X, Wang YM, Guo HJ, Chu S (2016). Ursodeoxycholic acid and 4-phenylbutyrate prevent endoplasmic reticulum stress-induced podocyte apoptosis in diabetic nephropathy. Lab Invest.

[120] Nie Q, Luo X, Wang K, Ding Y, Jia S, Zhao Q (2024). Gut symbionts alleviate MASH through a secondary bile acid biosynthetic pathway. Cell.

[121] Cervello M, Augello G, Cocco L, Ratti S, Follo MY, Martelli AM (2024). The potential of the nutraceutical berberine in the treatment of hepatocellular carcinoma and other liver diseases such as NAFLD and NASH. Adv Biol Regul.

[122] Qiu Y, Kang N, Wang X, Yao Y, Cui J, Zhang X (2023). Loss of Farnesoid X receptor (FXR) accelerates dysregulated glucose and renal injury in db/db mice. PeerJ.

[123] Wang XX, Wang D, Luo Y, Myakala K, Dobrinskikh E, Rosenberg AZ (2018). FXR/TGR5 Dual Agonist Prevents Progression of Nephropathy in Diabetes and Obesity. J Am Soc Nephrol.

[124] Chavez-Talavera O, Tailleux A, Lefebvre P, Staels B (2017). Bile Acid Control of Metabolism and Inflammation in Obesity, Type 2 Diabetes, Dyslipidemia, and Nonalcoholic Fatty Liver Disease. Gastroenterology.

[125] Zhou H, Ma C, Wang C, Gong L, Zhang Y, Li Y (2021). Research progress in use of traditional Chinese medicine monomer for treatment of non-alcoholic fatty liver disease. Eur J Pharmacol.

[126] Seldin MM, Meng Y, Qi H, Zhu W, Wang Z, Hazen SL (2016). Trimethylamine N-Oxide Promotes Vascular Inflammation Through Signaling of Mitogen-Activated Protein Kinase and Nuclear Factor-kappaB. J Am Heart Assoc.

[127] Tang WH, Wang Z, Levison BS, Koeth RA, Britt EB, Fu X (2013). Intestinal microbial metabolism of phosphatidylcholine and cardiovascular risk. N Engl J Med.

[128] Ma SR, Tong Q, Lin Y, Pan LB, Fu J, Peng R (2022). Berberine treats atherosclerosis via a vitamine-like effect down-regulating Choline-TMA-TMAO production pathway in gut microbiota. Signal Transduct Target Ther.

[129] Li DY, Tang WHW (2017). Gut Microbiota and Atherosclerosis. Curr Atheroscler Rep.

[130] Zeisel SH, Warrier M (2017). Trimethylamine N-Oxide, the Microbiome, and Heart and Kidney Disease. Annu Rev Nutr.

[131] Huang Y, Zhu Z, Huang Z, Zhou J (2023). Elevated serum trimethylamine oxide levels as potential biomarker for diabetic kidney disease. Endocr Connect.

[132] Fang Q, Zheng B, Liu N, Liu J, Liu W, Huang X (2021). Trimethylamine N-Oxide Exacerbates Renal Inflammation and Fibrosis in Rats With Diabetic Kidney Disease. Front Physiol.

[133] Leon-Mimila P, Villamil-Ramirez H, Li XS, Shih DM, Hui ST, Ocampo-Medina E (2021). Trimethylamine N-oxide levels are associated with NASH in obese subjects with type 2 diabetes. Diabetes Metab.

[134] Tan X, Liu Y, Long J, Chen S, Liao G, Wu S (2019). Trimethylamine N-Oxide Aggravates Liver Steatosis through Modulation of Bile Acid Metabolism and Inhibition of Farnesoid X Receptor Signaling in Nonalcoholic Fatty Liver Disease. Mol Nutr Food Res.

[135] Chen S, Henderson A, Petriello MC, Romano KA, Gearing M, Miao J (2019). Trimethylamine N-Oxide Binds and Activates PERK to Promote Metabolic Dysfunction. Cell Metab.

[136] Nagai Y, Akashi S, Nagafuku M, Ogata M, Iwakura Y, Akira S (2002). Essential role of MD-2 in LPS responsiveness and TLR4 distribution. Nat Immunol.

[137] Velasquez OR, Henninger K, Fowler M, Tso P, Crissinger KD (1993). Oleic acid-induced mucosal injury in developing piglet intestine. Am J Physiol.

[138] Cani PD, Amar J, Iglesias MA, Poggi M, Knauf C, Bastelica D (2007). Metabolic endotoxemia initiates obesity and insulin resistance. Diabetes.

[139] Pirlich M, Norman K, Lochs H, Bauditz J (2006). Role of intestinal function in cachexia. Curr Opin Clin Nutr Metab Care.

[140] Manco M, Putignani L, Bottazzo GF (2010). Gut microbiota, lipopolysaccharides, and innate immunity in the pathogenesis of obesity and cardiovascular risk. Endocr Rev.

[141] Li F, Yang N, Zhang L, Tan H, Huang B, Liang Y (2010). Increased expression of toll-like receptor 2 in rat diabetic nephropathy. Am J Nephrol.

[142] Lin JR, Wang ZT, Sun JJ, Yang YY, Li XX, Wang XR (2022). Gut microbiota and diabetic kidney diseases: Pathogenesis and therapeutic perspectives. World J Diabetes.

[143] Song YM, Lee YH, Kim JW, Ham DS, Kang ES, Cha BS (2015). Metformin alleviates hepatosteatosis by restoring SIRT1-mediated autophagy induction via an AMP-activated protein kinase-independent pathway. Autophagy.

[144] Han J, Wang Y (2018). mTORC1 signaling in hepatic lipid metabolism. Protein Cell.

[145] Gosis BS, Wada S, Thorsheim C, Li K, Jung S, Rhoades JH (2022). Inhibition of nonalcoholic fatty liver disease in mice by selective inhibition of mTORC1. Science.

[146] Fiorucci S, Distrutti E, Carino A, Zampella A, Biagioli M (2021). Bile acids and their receptors in metabolic disorders. Prog Lipid Res.

[147] Han SY, Song HK, Cha JJ, Han JY, Kang YS, Cha DR (2021). Farnesoid X receptor (FXR) agonist ameliorates systemic insulin resistance, dysregulation of lipid metabolism, and alterations of various organs in a type 2 diabetic kidney animal model. Acta Diabetol.

[148] Dionysopoulos G, Kalopitas G, Vadarlis A, Bakaloudi DR, Gkiourtzis N, Karanika E (2023). Can omega-3 fatty acids be beneficial in pediatric NAFLD?. A systematic review and meta-analysis. Crit Rev Food Sci Nutr.

[149] Stroes ES, Thompson PD, Corsini A, Vladutiu GD, Raal FJ, Ray KK (2015). Statin-associated muscle symptoms: impact on statin therapy-European Atherosclerosis Society Consensus Panel Statement on Assessment, Aetiology and Management. Eur Heart J.

[150] Maki KC, Ridker PM, Brown WV, Grundy SM, Sattar N, The Diabetes Subpanel of the National Lipid Association Expert P (2014). An assessment by the Statin Diabetes Safety Task Force: 2014 update. J Clin Lipidol.

[151] Preiss D, Seshasai SR, Welsh P, Murphy SA, Ho JE, Waters DD (2011). Risk of incident diabetes with intensive-dose compared with moderate-dose statin therapy: a meta-analysis. JAMA.

[152] Geng Q, Ren J, Song J, Li S, Chen H (2014). Meta-analysis of the effect of statins on renal function. Am J Cardiol.

[153] Dormuth CR, Hemmelgarn BR, Paterson JM, James MT, Teare GF, Raymond CB (2013). Use of high potency statins and rates of admission for acute kidney injury: multicenter, retrospective observational analysis of administrative databases. BMJ.

[154] Su X, Zhang L, Lv J, Wang J, Hou W, Xie X (2016). Effect of Statins on Kidney Disease Outcomes: A Systematic Review and Meta-analysis. Am J Kidney Dis.

[155] Newman CB, Preiss D, Tobert JA, Jacobson TA, Page RL 2nd, Goldstein LB (2019). Statin Safety and Associated Adverse Events: A Scientific Statement From the American Heart Association. Arterioscler Thromb Vasc Biol.

[156] Ward NC, Watts GF, Eckel RH (2019). Response by Ward et al to Letter Regarding Article, "Statin Toxicity: Mechanistic Insights and Clinical Implications". Circ Res.

[157] Wang Y, Zhan S, Du H, Li J, Khan SU, Aertgeerts B (2022). Safety of ezetimibe in lipid-lowering treatment: systematic review and meta-analysis of randomised controlled trials and cohort studies. BMJ Medicine.

[158] Thompson PD, Panza G, Zaleski A, Taylor B (2016). Statin-Associated Side Effects. J Am Coll Cardiol.

[159] Virani SS (2022). The Fibrates Story - A Tepid End to a PROMINENT Drug. N Engl J Med.

[160] Jiang T, Wang XX, Scherzer P, Wilson P, Tallman J, Takahashi H (2007). Farnesoid X receptor modulates renal lipid metabolism, fibrosis, and diabetic nephropathy. Diabetes.

[161] Yonezawa S, Kawasaki Y, Natori Y, Sugiyama A (2023). Improvement of LXR-mediated lipid metabolism in nephrotic model kidney accompanied by suppression of inflammation and fibrosis. Biochem Biophys Res Commun.

[162] Zhang Y, Zhang X, Chen L, Wu J, Su D, Lu WJ (2006). Liver X receptor agonist TO-901317 upregulates SCD1 expression in renal proximal straight tubule. Am J Physiol Renal Physiol.

[163] Zhong Y, Lee K, Deng Y, Ma Y, Chen Y, Li X (2019). Arctigenin attenuates diabetic kidney disease through the activation of PP2A in podocytes. Nat Commun.

[164] Li P, Chen Y, Liu J, Hong J, Deng Y, Yang F (2015). Efficacy and safety of tangshen formula on patients with type 2 diabetic kidney disease: a multicenter double-blinded randomized placebo-controlled trial. PLoS One.

[165] Zhao J, Tostivint I, Xu L, Huang J, Gambotti L, Boffa JJ (2022). Efficacy of Combined Abelmoschus manihot and Irbesartan for Reduction of Albuminuria in Patients With Type 2 Diabetes and Diabetic Kidney Disease: A Multicenter Randomized Double-Blind Parallel Controlled Clinical Trial. Diabetes Care.

[166] Yao Y, Yu YC, Cai MR, Zhang ZQ, Bai J, Wu HM (2022). UPLC-MS/MS method for the determination of the herb composition of Tangshen formula and the *in vivo* pharmacokinetics of its metabolites in rat plasma. Phytochem Anal.

[167] Wang Y, Zhao H, Li X, Li N, Wang Q, Liu Y (2019). Tangshen Formula Alleviates Hepatic Steatosis by Inducing Autophagy Through the AMPK/SIRT1 Pathway. Front Physiol.

[168] Liu P, Peng L, Zhang H, Tang PM, Zhao T, Yan M (2018). Tangshen Formula Attenuates Diabetic Nephropathy by Promoting ABCA1-Mediated Renal Cholesterol Efflux in db/db Mice. Front Physiol.

[169] Kong Q, Zhang H, Zhao T, Zhang W, Yan M, Dong X (2016). Tangshen formula attenuates hepatic steatosis by inhibiting hepatic lipogenesis and augmenting fatty acid oxidation in db/db mice. Int J Mol Med.

[170] Zhao H, Li X, Zhao T, Zhang H, Yan M, Dong X (2017). Tangshen formula attenuates diabetic renal injuries by upregulating autophagy via inhibition of PLZF expression. PLoS One.

[171] Katsiki N, Mikhailidis DP, Mantzoros CS (2016). Non-alcoholic fatty liver disease and dyslipidemia: An update. Metabolism.

[172] Mitrofanova A, Burke G, Merscher S, Fornoni A (2021). New insights into renal lipid dysmetabolism in diabetic kidney disease. World J Diabetes.

[173] Chen P, Zhao J, Zhang H, Yang X, Zhao T, Zhang H (2017). Tangshen Formula Attenuates Colonic Structure Remodeling in Type 2 Diabetic Rats. Evid Based Complement Alternat Med.

[174] Zhao T, Zhang H, Yin X, Zhao H, Ma L, Yan M (2020). Tangshen formula modulates gut Microbiota and reduces gut-derived toxins in diabetic nephropathy rats. Biomed Pharmacother.

[175] Huang L, Zhang Y, Zhang X, Chen X, Wang Y, Lu J (2019). Therapeutic Potential of Pien-Tze-Huang: A Review on Its Chemical Composition, Pharmacology, and Clinical Application. Molecules.

[176] Zeng X, Zhang X, Su H, Gou H, Lau HC-H, Hu X (2024). Pien Tze Huang Protects Against Non-Alcoholic Steatohepatitis by Modulating the Gut Microbiota and Metabolites in Mice. Engineering.

[177] Zhang J, Cao G, Tian L, Hou J, Zhang Y, Xu H (2023). Intragastric administration of Pien Tze Huang enhanced wound healing in diabetes by inhibiting inflammation and improving energy generation. Phytomedicine.

[178] Li Y, Yao J, Han C, Yang J, Chaudhry MT, Wang S (2016). Quercetin, Inflammation and Immunity. Nutrients.

[179] Gnoni A, Di Chiara Stanca B, Giannotti L, Gnoni GV, Siculella L, Damiano F (2022). Quercetin Reduces Lipid Accumulation in a Cell Model of NAFLD by Inhibiting *De Novo* Fatty Acid Synthesis through the Acetyl-CoA Carboxylase 1/AMPK/PP2A Axis. Int J Mol Sci.

[180] Jiang X, Yu J, Wang X, Ge J, Li N (2019). Quercetin improves lipid metabolism via SCAP-SREBP2-LDLr signaling pathway in early stage diabetic nephropathy. Diabetes Metab Syndr Obes.

[181] Pang B, Zhao LH, Zhou Q, Zhao TY, Wang H, Gu CJ (2015). Application of berberine on treating type 2 diabetes mellitus. Int J Endocrinol.

[182] Koperska A, Wesolek A, Moszak M, Szulinska M (2022). Berberine in Non-Alcoholic Fatty Liver Disease-A Review. Nutrients.

[183] Qin X, Zhao Y, Gong J, Huang W, Su H, Yuan F (2019). Berberine Protects Glomerular Podocytes via Inhibiting Drp1-Mediated Mitochondrial Fission and Dysfunction. Theranostics.

[184] Wang Y, Tong Q, Shou JW, Zhao ZX, Li XY, Zhang XF (2017). Gut Microbiota-Mediated Personalized Treatment of Hyperlipidemia Using Berberine. Theranostics.

[185] Hoseini A, Namazi G, Farrokhian A, Reiner Ž, Aghadavod E, Bahmani F (2019). The effects of resveratrol on metabolic status in patients with type 2 diabetes mellitus and coronary heart disease. Food & Function.

[186] Chen XX, Xu YY, Wu R, Chen Z, Fang K, Han YX (2019). Resveratrol Reduces Glucolipid Metabolic Dysfunction and Learning and Memory Impairment in a NAFLD Rat Model: Involvement in Regulating the Imbalance of Nesfatin-1 Abundance and Copine 6 Expression. Front Endocrinol (Lausanne).

[187] Gu W, Wang X, Zhao H, Geng J, Li X, Zheng K (2022). Resveratrol ameliorates diabetic kidney injury by reducing lipotoxicity and modulates expression of components of the junctional adhesion molecule-like/sirtuin 1 lipid metabolism pathway. Eur J Pharmacol.

[188] Li S, Shi Y, Liu P, Song Y, Liu Y, Ying L (2020). Metformin inhibits intracranial aneurysm formation and progression by regulating vascular smooth muscle cell phenotype switching via the AMPK/ACC pathway. J Neuroinflammation.

[189] Plosch T, Kok T, Bloks VW, Smit MJ, Havinga R, Chimini G (2002). Increased hepatobiliary and fecal cholesterol excretion upon activation of the liver X receptor is independent of ABCA1. J Biol Chem.

[190] Qin X, Jiang M, Zhao Y, Gong J, Su H, Yuan F (2020). Berberine protects against diabetic kidney disease via promoting PGC-1alpha-regulated mitochondrial energy homeostasis. Br J Pharmacol.

[191] Zhang Z, Zhang H, Li B, Meng X, Wang J, Zhang Y (2014). Berberine activates thermogenesis in white and brown adipose tissue. Nat Commun.

[192] Sadi G, Pektas MB, Koca HB, Tosun M, Koca T (2015). Resveratrol improves hepatic insulin signaling and reduces the inflammatory response in streptozotocin-induced diabetes. Gene.

[193] Lee M, Katerelos M, Gleich K, Galic S, Kemp BE, Mount PF (2018). Phosphorylation of Acetyl-CoA Carboxylase by AMPK Reduces Renal Fibrosis and Is Essential for the Anti-Fibrotic Effect of Metformin. J Am Soc Nephrol.

[194] Yonamine CY, Pinheiro-Machado E, Michalani ML, Freitas HS, Okamoto MM, Correa-Giannella ML (2016). Resveratrol improves glycemic control in insulin-treated diabetic rats: participation of the hepatic territory. Nutr Metab (Lond).

[195] Du L, Hao M, Li C, Wu W, Wang W, Ma Z (2017). Quercetin inhibited epithelial mesenchymal transition in diabetic rats, high-glucose-cultured lens, and SRA01/04 cells through transforming growth factor-beta2/phosphoinositide 3-kinase/Akt pathway. Mol Cell Endocrinol.

[196] Pisonero-Vaquero S, Martinez-Ferreras A, Garcia-Mediavilla MV, Martinez-Florez S, Fernandez A, Benet M (2015). Quercetin ameliorates dysregulation of lipid metabolism genes via the PI3K/AKT pathway in a diet-induced mouse model of nonalcoholic fatty liver disease. Mol Nutr Food Res.

